# In vivo redox imaging of plasma-induced skin-inflammation in mice

**DOI:** 10.1038/s44303-024-00029-z

**Published:** 2024-08-02

**Authors:** Yassien Badr, Abdelazim Elsayed Elhelaly, Fuminori Hyodo, Koki Ichihashi, Hiroyuki Tomita, Yoshifumi Noda, Hiroki Kato, Masayuki Matsuo

**Affiliations:** 1 Department of Radiology, Frontier Science for Imaging, Gifu University, Gifu, Japan; 2 Department of Infectious Diseases and Epidemics, Faculty of Veterinary Medicine, Damanhour University, El-Beheira, Egypt; 3 Faculty of Veterinary Medicine, Suez Canal University, Ismailia, Egypt; 4 Department of Pharmacology, Graduate School of Medicine, Gifu University, Gifu, Japan; 5 Center for One Medicine Innovative Translational Research (COMIT), Gifu University, Gifu, Japan; 6 Innovation Research Center for Quantum Medicine, Gifu University, Gifu, Japan; 7 Department of Tumor Pathology, Gifu University Graduate School of Medicine, Gifu, Japan; 8 Department of Radiology, Gifu University, Gifu, Japan

**Keywords:** Biochemistry, Chemical biology, Health care, Medical imaging

## Abstract

Cold atmospheric plasma (CAP) generates reactive oxygen species (ROS) which induce biological effects on living cells. CAP has potential applications in medicine, but its highly reactive nature can lead to adverse skin complications. A noninvasive technique to examine redox changes in skin is needed for monitoring the treatment process. This study was conducted to develop a skin-inflammation model triggered by CAP-derived ROS and to monitor its progression noninvasively by in vivo dynamic nuclear polarization-MRI (DNP-MRI). The model was successfully developed by exposing the skin to both direct and remote modes of CAP. In vivo DNP-MRI imaging revealed faster reduction rates of TEMPOL in plasma-irradiated skin-inflammation areas, particularly in the remote mode plasma-irradiated skin. MRI revealed high-intensity areas in both the superficial and deep layers of the plasma-irradiated skin. The study highlights the potential importance of DNP-MRI in imaging skin-inflammation models and could improve the use of CAP in medical treatments.

## Introduction

Plasma medicine is a rapidly growing field that includes physics, chemistry, biology, and medicine. Physical plasma can be considered as the fourth state of matter and is generated by supplying energy to a gas medium to induce its ionization, excitation, or dissociation. When these ionized or excited gaseous molecules, atoms, electrons, and photons interact with the target surfaces, they induce production of free radicals with biological potential and a thermal effect^1–3^. Depending on the relative temperatures of the ions and uncharged molecules, plasmas are divided into thermal (hot) plasma and nonthermal (cold) plasma^4^. Cold atmospheric plasma (CAP) generates ions and radicals at room temperature that can be used in several biomedical applications. Clinical research in plasma medicine has mainly focused on dermatological applications for tissue regeneration and wound healing^5,6^. However, the emerging clinical potential of CAP has revolutionarily expanded to include sterilization of surfaces and living tissues^4^, blood coagulation^7^, antibacterial activity^4,8,9^, stimulation of cell permeabilization for transfection^10^, and cancer treatment^1,11,12^.

Although CAP is receiving increased attention because of its potential applications in a variety of fields, including skin-care medicine, its application to skin has implications^13^ that must be considered, including potential skin damage and inflammation to more serious conditions^14^, especially when treatment times and gas flow rates are not properly controlled. Various mechanisms are involved in these adverse effects of CAP, including the sensitivity that patients may experience, thermal effects, UV radiation damage, and the generated reactive oxygen species (ROS). Therefore, understanding and monitoring the adverse effects of CAP on skin is critical to ensure safe and effective applications in dermatological and cosmetic procedures.

The main biologically active components generated by CAP irradiation are ROS and reactive nitrogen species (RNS) or jointly named (RONS), with ROS recognized as the primary active agents^15^. Plasma-generated ROS can induce biological effects by irradiating CAP directly or indirectly on cells/tissue^1,2^. Other physical components generated by plasma (UV photons and electromagnetic fields) appear to have negligible cellular influence by themselves^16^. The mechanistic actions underlying the effects of plasma-derived ROS on living cells/tissue are believed to be mediated mainly through oxidative stress in treated biotargets. Oxidative alterations induce various biological and physiological pathways, such as cell-membrane signaling, gene expression and epigenetic changes, endocrine pathways, effects on the cell cycle, and apoptosis^1^. Large amounts of plasma induce certain effects, whereas small amounts have different effects on biological systems. Consequently, one of the primary concerns in plasma medicine is standardizing the irradiation process by adjusting the type, amount of reactive species, and exposure time to achieve a particular therapeutic or biological effect^2^. Among ROS, hydroxyl (OH) radicals are of special importance because of their high reactivity and higher oxidative potential; they are strongly oxidizing and may negatively affect a wide spectrum of organic substances, making them particularly relevant in the context of oxidative stress and its health consequences^17^. This high reactivity and oxidative potential make OH radicals a primary focus in the study of oxidative stress and its effect on health status.

A drawback of CAP is the damage it causes to treated skin. This damage is caused by thermal effects, UV radiation, or the generated ROS^18^. Consensus guidelines state that ROS-mediated cell death is a type of controlled cell death^19^, which also has shown a link between ROS and a variety of cell death pathways, including intrinsic apoptosis, necrosis, necroptosis, and autophagy, owing to the role of ROS throughout metabolism, mitochondrial homeostasis, inflammation, and immunology^2^.

Noninvasive imaging techniques, such as magnetic resonance imaging (MRI) or in vivo DNP-MRI, have not previously been used to investigate skin damage induced mainly by cold plasma-derived OH radicals. Therefore, noninvasive evaluation of the level of skin damage after direct and indirect plasma irradiation over time would be useful. Of particular interest is the skin-inflammation model established by continuous exposure to OH radicals produced by CAP. Mouse skin was selected as a model to induce and evaluate possible skin damage. We used in vivo DNP-MRI, a technique we developed for this purpose, to monitor redox changes in vivo in the skin of mice used as the animal model. In this technique, the MRI signal is improved by exciting free-radical probes by irradiation of the target tissue at their electron paramagnetic resonance (EPR) frequency. Nitroxide as a redox-sensitive agent is typically used in DNP-MRI as the probe in this method^20–22^. Nitroxides are redox sensitive compounds that undergo a series of transformations in the presence of free radicals, eventually resulting in their reduction. When exposed to free radicals, nitroxides are transformed into oxoammonium cations. This reaction includes transferring an electron from the nitroxide to the free radical. These oxoammonium cations are then further reduced into hydroxylamines in the presence of a reducing agent, such as NAD(P)H. This reduction process is of significant value because it highlights the redox sensitivity and the critical role of nitroxides in redox reactions, as well as their ability to act as antioxidants^23,24^.

The aim of this study was to visualize free radicals generated by direct and remote CAP applied in various in vitro assays with different exposure durations. We also investigated the possibility of in vivo noninvasive imaging by MRI and in vivo DNP-MRI of the redox status of skin and subcutaneous layers following exposure to ROS, especially OH radicals, generated by plasma irradiation in a plasma-induced mouse skin-inflammation model, which to our knowledge has not been reported previously.

## Results

### EPR experiments using DMPO demonstrate the feasibility of measuring plasma-generated OH radicals

**Fig. 1:**
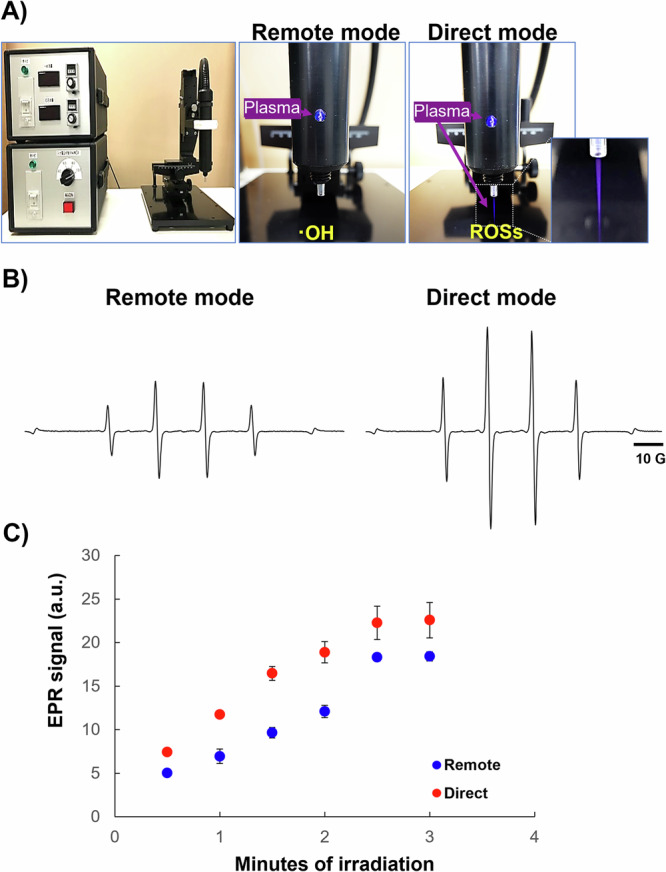
Demonstration of the plasma-generating machine and EPR spectroscopy of plasma-irradiated DMPO. **A** The entire machine (left). Modes of the plasma-generating machine and the free radicals producible. **B** *X*-band EPR spectra of DMPO-OH induced by 2 min of either remote or direct plasma irradiation into a 10-mM DMPO solution. **C** Irradiation time-dependent production of the DMPO-OH signal by plasma. The signal areas of the DMPO-OH spectra induced by irradiation with plasma (direct or remote) into 10-mM DMPO solution for different periods were estimated (*n* = 3).

DMPO solution was used in both the direct and remote modes of the instrument to compare the generation of OH radicals after plasma irradiation. DMPO is a spin-trapping agent that can bind the OH radical and increase its half-life, which enables EPR detection and quantification by detecting the DMPO-OH specific spectrum^25,26^. The typical four-peak spectra of DMPO-OH was observed, and the intensities of these peaks were higher in the direct mode of plasma irradiation than in the remote mode (Fig. 1B). In addition, the signal intensities in both modes increased steadily as the irradiation periods increased (Fig. 1C). In all the tested irradiation periods, signal intensities were higher in the direct mode than in the remote mode (Fig. 1C). These results indicate that plasma-generated OH radicals were more abundant in the direct mode. Moreover, the generation of OH radicals was proven to be proportional to the irradiation time.

To ascertain whether the OH radicals identified using DMPO are the primary radicals produced by plasma irradiation, another spin trapping experiment was conducted utilizing superoxide dismutase (SOD) and catalase. Plasma irradiation (remote or direct mode) was conducted on a DMPO solution mixed with SOD, catalase, or both, and the EPR signals were obtained and compared to those of the DMPO solution only. It was observed that the addition of SOD had a less pronounced effect on the production of DMPO-OH in both modes. Conversely, the addition of catalase had a more pronounced effect on the production of DMPO-OH (Fig. S1). These results may indicate that less superoxide radicals and more hydrogen peroxide are formed in the solution during the plasma irradiation.

### DNP-MRI of ROS-induced redox reaction after direct- and remote mode CAP exposure

**Fig. 2:**
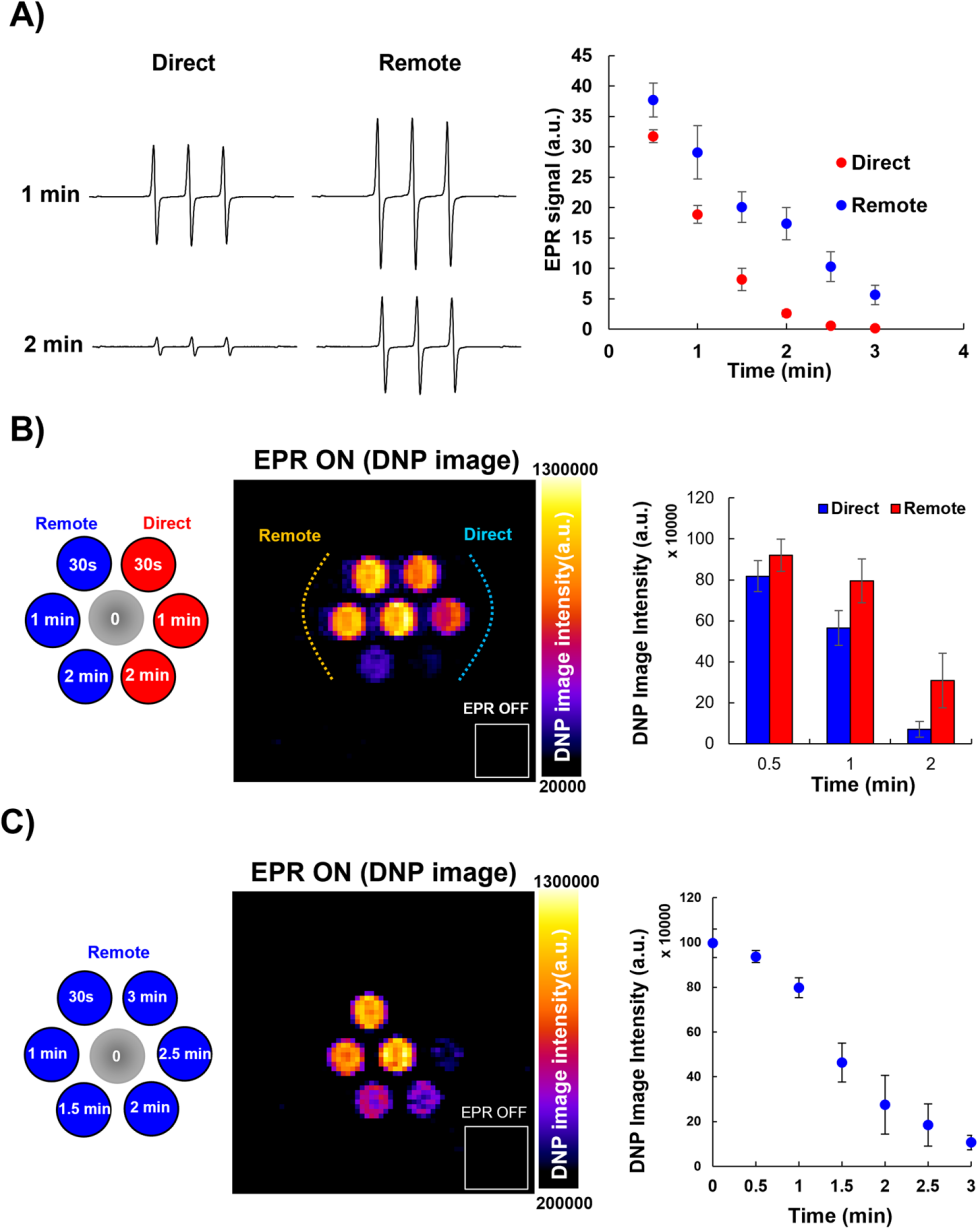
EPR analysis and in vitro DNP-MRI imaging of plasma-irradiated TEMPOL solution (1-mM TEMPOL with 2-mM glutathione). **A** TEMPOL spectra after plasma irradiation, direct or remote modes, for 1 and 2 min (left). Signal areas of TEMPOL spectra induced by irradiation with either direct or remote modes for different periods; *n* = 3 (right). **B** DNP-MRI phantom imaging of TEMPOL solutions irradiated with plasma (direct or remote) for three different times (the central tube was not irradiated) (left). Image intensities in each tube when EPR is ON and OFF (middle). Comparison of image intensities in either direct or remote modes of irradiated tubes for three different times; *n* = 3 (right). **C** DNP-MRI phantom imaging of TEMPOL solutions irradiated with remote mode plasma for six different times of exposure (the central tube was not irradiated) (left). Image intensities in each tube when EPR is ON and OFF (middle). Comparison of the image intensities of tubes irradiated for different times; *n* = 3 (right).

To test the feasibility of monitoring the redox reactions that can occur by CAP-produced ROS using DNP-MRI, a solution of 1-mM TEMPOL + 2-mM GSH was irradiated with plasma for 1 or 2 min in direct and remote modes, and the irradiated solution was examined by EPR. The intensity of the TEMPOL spectra was higher in the remote mode irradiated samples than in the direct mode after both irradiation periods (Fig. 2A, left), indicating a higher rate of TEMPOL reduction in the direct mode. This result indicates that more ROS are generated in the direct mode. Furthermore, signal intensities decreased steadily as the irradiation time increased (Fig. 2A, right), indicating a higher rate of TEMPOL reduction due to more ROS generated after longer irradiation times. Signal intensities were lower in the direct mode than in the remote mode across all irradiation periods (Fig. 2A, right), indicating a higher rate of TEMPOL reduction and higher ROS production in the direct mode than in the remote mode.

To compare DNP-MRI image enhancement with EPR irradiation (EPR ON) after plasma irradiation, we irradiated phantoms filled with TEMPOL + GSH solution in the remote or direct modes. Using equal irradiation periods, we found that image intensities were higher in the remote mode irradiated wells than in the direct mode irradiated ones, indicating a higher reduction rate of TEMPOL in the direct mode (Fig. 2B, left). Furthermore, image intensities decreased linearly with increasing irradiation time (Fig. 2B, right), indicating that more ROS were generated. These results are consistent with those of previous experiments using plasma-irradiated DMPO solutions (Fig. 1B, C).

Next, we investigated the relationship between irradiation time and DNP-MRI enhancement by comparing different periods of remote mode plasma irradiation time. The results showed that the intensity of the images decreased steadily as the irradiation time increased (Fig. 2C), indicating a higher rate of TEMPOL reduction due to the higher ROS generation during the longer exposure to plasma.

### Plasma irradiation induces skin-inflammation and damage

**Fig. 3:**
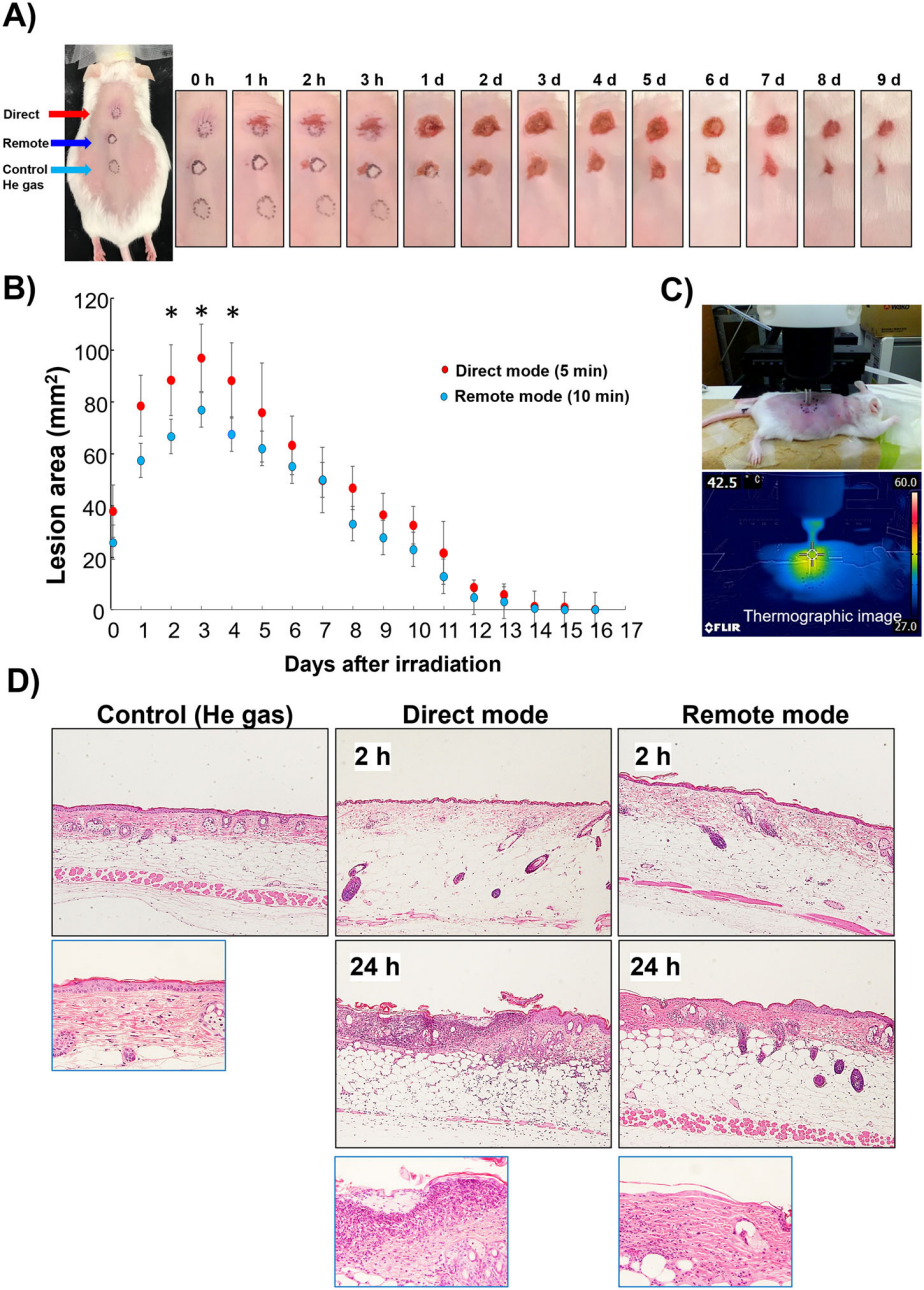
Mouse skin-inflammation model induced by plasma irradiation. **A** Three different sites were chosen on the shaved mouse back: the upper site was irradiated with direct mode plasma for 5 min, the middle was irradiated with remote mode plasma for 10 min, and the lower was irradiated with helium gas only for 10 min. The animal’s back was photographed immediately after irradiation (0 h) and at various time intervals to demonstrate the lesion’s progression. **B** Progression of the skin injury area after plasma irradiation until complete healing, occurring 16 days after irradiation (*n* = 4 per group). The (*) indicates statistically significant differences between the two groups at the *p* < 0.05 level, as determined by a one-tailed *t*-test assuming equal variance. **C** Thermography measurement: skin-surface temperature was measured after 10-min of plasma irradiation in remote mode. The skin-surface temperature was 42.5 °C. **D** Histopathology of skin samples irradiated by either remote or direct plasma irradiation, collected at 2 and 24 h after irradiation, fixed, and examined by regular histopathological procedures.

In our murine model of plasma-induced skin-inflammation, redness and swelling in and around the irradiated areas were observed immediately after CAP irradiation. The affected skin areas increased over time after plasma treatment followed by sloughing of the superficial skin layers leaving fresh wounds, reaching maximum lesion areas on the third day after irradiation. Later, the injury area of each wound declined gradually until complete healing at 2 weeks post-irradiation (Fig. 3A, B), leaving scar tissue at the injured site.

In our experiment, the areas of skin injuries induced by direct mode plasma irradiation were noticeably larger than those caused by remote mode plasma (Fig. 3A, B). The differences in the lesion size were significantly larger at 2-, 3-, and 4-days post-irradiation (Fig. 3B). To exclude the possibility that the resulting effect was caused by the increasing temperature during plasma irradiation rather than by the free radicals, we used a thermography camera to measure the skin-surface temperature and found that it was always <42.5 °C, which does not cause burns (Fig. 3C).

Histopathological investigation of skin lesions collected from plasma-irradiated skin at both 2 and 24 h after irradiation revealed that epidermal damage and loss were milder in the remote mode irradiated samples than in the direct mode irradiated ones. We also observed less inflammation and fibrosis in the remote mode than in the direct mode irradiated skin samples (Fig. 3D).

### Plasma-induced skin-inflammation model monitored by in vivo DNP-MRI

**Fig. 4:**
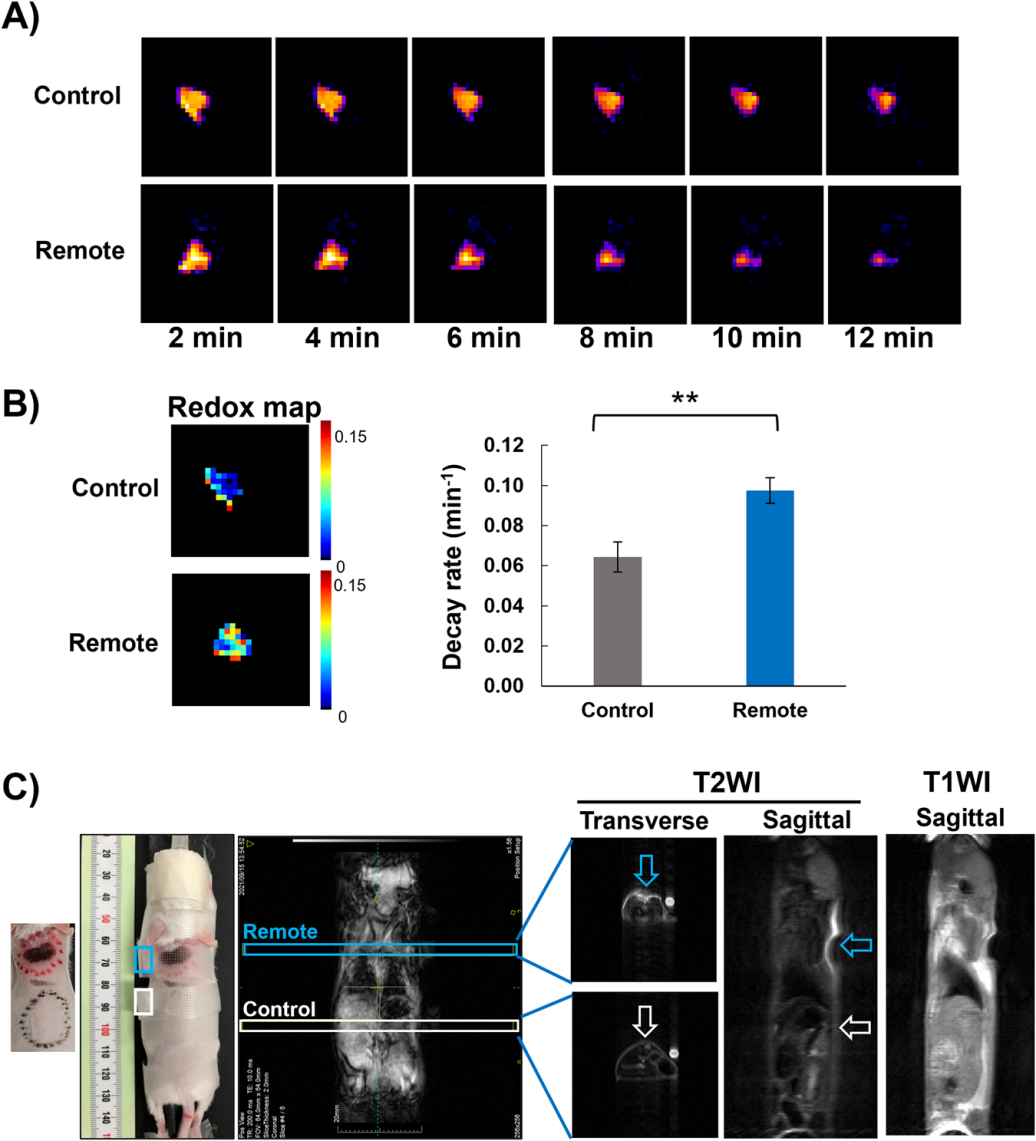
In vivo DNP-MRI and conventional MRI of remote mode plasma-induced skin-inflammation compared with control skin (images were taken 24 h after plasma irradiation). **A** In vivo DNP-MRI pharmacokinetic imaging of plasma irradiation-induced skin-inflammation and control skin. DNP-MRI images were captured over time following subcutaneous injection of 150 µl TEMPOL (of 2.5 mM concentration) under inflamed- skin or control regions. **B** Redox maps were calculated from the pharmacokinetic DNP images of the inflamed skin and control regions. The reduction rate of each pixel on the four pharmacokinetics in vivo DNP-MRI images was calculated and visualized as a redox map (left). Comparison of decay rates of DNP image intensity in the inflamed skin and control regions. Decay rates were calculated by the slope of image intensity in the region of interest corresponding to the enhancement by TEMPOL; *n* = 5 per group. The *t*-test was used to assess the results, **P < 0.01 (right). **C** In vivo conventional MRI in a plasma-irradiated mouse (inflamed skin, surrounded by a red circle; and control skin surrounded by a black circle on the left). TEMPOL solution-containing phantoms were used as a control for intensity and put next to the plasma-irradiated and control skin (blue and white small rectangulars, respectively). Transverse sections of the irradiated and control skins are shown. In addition, a sagittal section involving the entire mouse body is shown.

The in vivo redox status of remote mode plasma-irradiated skin was compared with that of the control skin. TEMPOL was subcutaneously injected under the plasma-irradiated and control skin, and DNP-MRI images were then obtained at fixed time points while EPR was ON. The enhancement of image intensity faded gradually over time (Fig. 4A). The image intensity decayed faster in the plasma-irradiated skin than in the control skin (Fig. 4A, B right), indicating a higher reduction rate of TEMPOL in the plasma-treated skin, which suggests higher levels of RONS. Furthermore, redox maps revealed a higher reduction rate in the plasma-irradiated skin than in the control skin (Fig. 4B, left). These findings suggest that oxidative stress in plasma-irradiated skin is related to the rapid decay and higher reduction rates of TEMPOL.

### Anatomical imaging by conventional MRI at clinical field strength shows skin damage after plasma treatment

MRI was used to investigate the possibility of visualizing plasma-induced skin-inflammation in vivo. A mouse skin region was irradiated with remote mode plasma as described above, and a sagittal section of the whole mouse as well as two transverse sections, one from the irradiated skin and the other from the control skin, were examined after 24 h. Compared with the control skin site, the plasma-irradiated skin site showed high-intensity areas in both the sagittal and transverse sections (Fig. 4C) indicating inflamed skin tissues. Areas of deeper skin damage were also observable in the skin samples treated with the remote mode plasma.

## Discussion

Plasma medicine is an emerging field that includes physics, biology, and medicine, with promising therapeutic applications for various pathological conditions. The biomedical effects of CAP are mediated mainly through the generated reactive species that contact the biotargets. Plasma-generated RONS can be useful and provide insights for studying redox biology^27^; on the other hand, studies using plasma in redox biology will also serve the field of plasma medicine. Therefore, advancements in both redox biology and plasma medicine are mutually beneficial.

Since the biological effects of plasma are mediated in principle through its redox modifications in the target tissues, a noninvasive method that enables in vivo monitoring of redox changes that follow plasma treatment of live tissues will be of high value in monitoring and/or evaluating the treatment response after plasma irradiation.

DMPO, the spin-trapping agent, and EPR spectroscopy revealed that OH radicals produced by plasma irradiation were directly related to the time of exposure and were higher in the direct mode. These findings are consistent with previous findings^25^. The addition of catalase to the DMPO solution followed by plasma irradiation and EPR spectroscopy demonstrated that hydrogen peroxide is also produced in substantial quantities in the solution during plasma exposure. However, hydrogen peroxide is a relatively stable molecule that can also act as a precursor to the highly reactive hydroxyl radical which typically occurs via the Fenton reaction in vivo^28^.

DNP-MRI, an imaging tool for detecting redox status, was successfully used for imaging the ROS-induced redox reaction shifts induced by CAP irradiation of the TEMPOL/GSH mixture solution. The results also confirmed that the higher rates of ROS generated in the direct mode were proportionally dependent on the exposure time.

We succeeded in developing a murine skin-inflammation model to study the potential adverse effects of the OH radicals and the other ROS produced in the remote mode of CAP irradiation. Several methods are being used to examine the adverse effects of CAP on the skin. These methods include histopathological examination^14,29^, temperature monitoring of the skin by thermal cameras or probes during plasma treatment^29,30^, clinical observations, surveys, and self-reports by patients undergoing CAP for any negative effects that might be noticed^31^ as well as imaging tools, such as electron spectroscopic analysis, infrared spectroscopy, or confocal microscopy^32^, to visualize and analyze changes in skin structure, hydration levels, and cellular activity after plasma exposure. However, our study revealed that MRI and in vivo DNP-MRI techniques were much more comprehensive in studying this model. In vivo DNP-MRI was especially useful for noninvasively monitoring the redox status of the subcutaneous layers under the affected skin-surface. MRI at clinical field strength also proved useful for monitoring skin damage that may result from the medical application of plasma, without the need for any contrast agents.

We conducted a pilot experiment to establish a skin-inflammation model by plasma irradiation for 5 min in either direct or remote modes. In the direct mode, a 5-min exposure was sufficient to produce clear skin damage, but in the remote mode, a 10-min exposure was needed. Similar to a previous study’s findings^14^, our skin-inflammation model showed immediate redness and swelling in and around the irradiated areas, which continued and gradually healed until a complete recovery at 2 weeks after irradiation. In addition, the observed skin damage was proportional to the treatment time and plasma flow rate. These skin complications observed in our study and in previous studies may be attributed to the effects of RONS in addition to the physical effects of plasma^18^. However, in this study, we compared the remote and direct plasma-irradiation modes. The remote mode is characterized by the ability to minimize most of the physical, thermal, and UV effects of the direct mode. Therefore, we believe that the short-lived ROS and RNS (e.g., OH radicals, atomic oxygen, nitric oxide) produced by plasma are the main effectors in this condition. Moreover, the late skin damage that occurs after plasma irradiation (≥24 h after irradiation) has been referred to as “indirect damage” and is believed to be related to the diffusion of RONS^14^. Unlike direct damage, indirect damage was not related to plasma irradiation time or flow rate^14^. To the best of our knowledge, this is the first report of a skin-inflammation model induced mainly by the OH radicals of remote CAP exposure.

The damaging effect on skin was stronger in direct mode than in remote mode, not only due to the presence of higher amounts of ROS but also due to the direct physical and thermal effects of plasma on the skin. However, the effect was less severe in the remote mode, which mainly generates hydroxyl radicals with a minimum physical effect. These observations are supported by the in vitro experiments in our study that showed more free radicals were released from the direct mode plasma than from the remote mode, and by the skin-temperature measurements during plasma irradiation made by the thermography camera.

The damaging effects of plasma treatment on murine skin at the molecular and physiological levels may be attributed to alterations in the junctional network, increased disaggregation of cells in the stratum corneum, increased skin oxygenation and perfusion, and altered oxidation of skin lipids^33^.

DNP-MRI is an emerging biomedical technique that is used in noninvasive molecular and metabolic imaging^21^. Various imaging techniques have been used to visualize skin-inflammation models, including MRI, computed tomography, and ultrasound. However, DNP-MRI has gained significant attention recently because of its superior sensitivity and specificity in detecting the redox status of skin. Recent studies have used in vivo DNP-MRI to visualize the redox status of the skin and free radicals in atopic dermatitis skin lesions^34^. The current study confirmed that in vivo DNP-MRI technique is effectively useful for noninvasive imaging of skin-inflammation with high resolution and accuracy.

Future strategies should focus on improving the use of DNP-MRI for skin-inflammation imaging. These strategies include the use of different models, such as the irritant-contact dermatitis model or the allergic contact dermatitis model^35^. Furthermore, the development of higher sensitivity contrast agents for DNP-MRI that improve specificity in imaging of skin-inflammation could increase the application of this technique in this field.

In this study, we succeeded in developing a murine skin damage model using CAP irradiation. For the first time, we also succeeded in noninvasive visualization of the redox changes that occur in animal tissue caused by inflammation and damage that may follow the medical uses of CAP.

The findings of our study could aid in monitoring redox alterations after plasma treatment and in evaluating responses to irradiating plasma on live tissue, especially for therapeutic purposes. Our results also can support ongoing efforts to develop methods for adjusting plasma irradiation to produce a specific medical response/effect. Notably, the potential importance of DNP-MRI for imaging skin-inflammation models developed by exposure to the ROS, especially OH radicals, formed during remote CAP exposure was highlighted. Overall, it is hoped that the study findings will make a valuable contribution to ongoing advancements in the field of skin-inflammation imaging.

## Methods

### Animals

All animal care and experimental protocols were approved by the Committee for Animal Research and Welfare of the Medical School, Gifu University, and were conducted according to the recommendations of the Committee for Care and Use of Laboratory Animals, Gifu University. Male 6–8-week-old BALB/c mice weighing 20–25 g were purchased from Charles River Laboratories Japan, Inc. (Yokohama, Japan). Before the experiments, all animals were acclimated to a normal diet for 1 week with free access to distilled water and appropriate food (MF diet, Oriental Yeast Co., Tokyo, Japan). Every effort was made to minimize the number of mice used and their suffering.

### Chemicals

We purchased 5,5-dimethyl-1-pyrroline N-oxide (DMPO) from Dojindo Laboratories (Kumamoto, Japan). The chemicals 4-hydroxy-2,2,6,6-tetramethylpiperidin-1-oxyl (TEMPOL) and glutathione (GSH) were purchased from Tokyo Chemical Industry Co. Ltd. (Tokyo, Japan). All other chemicals were commercially available and of reagent-grade quality.

### Cold atmospheric helium-based plasma generation and treatment

A CAP system (SE-ASG, Seinan Industrial Co., Ltd., Osaka, Japan) with a helium gas flow controller, a voltage power supply controller, and a plasma jet was used to generate CAP at room temperature. The diameter of the dielectric tube was 2 mm. The plasma jet was 20 mm long for generating plasma with helium gas at a flow rate of 2 L/min. The discharge gap between the bottom of the quartz and the treated sample surface was fixed at 5 mm (Fig. 1A). Two different modes were supported by this instrument: the direct mode, which releases different types of ROS, and the remote mode, which produces mainly OH radicals. A photograph of the platform and the two different modes is shown in Fig. 1A. The mechanism of action has been previously described^3^.

### Measurement of OH radicals by *X*-band EPR

For the measurement of plasma-generated OH radicals, a 10-mM DMPO solution was irradiated with plasma, either in direct or remote mode. Because OH radicals have a very short half-life, their combination with DMPO (generating DMPO-OH) extends their half-life and allows their detection by different approaches^25^. Furthermore, different irradiation periods (0.5, 1, 1.5, 2, 2.5, and 3 min) were used to investigate the irradiation dependence of OH radical generation. The distance between the plasma nozzle and the DMPO solution surface was maintained at 5 mm. DMPO-OH spectra were detected 40 s after the end of plasma irradiation by *X-*band EPR spectrometry (JEOL Ltd. Tokyo, Japan). Measurements were made under the following conditions: room temperature, microwave power = 1 mW, microwave frequency = 9.4 Ghz (336 mT), modulation amplitude = 0.06 mT, sweep width = ±5 mT, time constant = 0.03 s, and sweep time = 1 min.

TEMPOL was used as a DNP contrast agent in DNP-MRI, and its spectra were investigated as previously stated^21^. Briefly, a TEMPOL-GSH solution (1 mM TEMPOL + 2 mM GSH) was irradiated with plasma (direct or remote) for different irradiation periods and then analyzed using an *X*-band EPR spectrometer.

### Phantom imaging using DNP-MRI

The DNP phenomenon is produced in DNP-MRI by induction of a resonant microwave at a frequency that matches the electron spin of the free-radical solution. For this purpose, a low-field DNP-MRI system (Keller) obtained from Japan Redox Ltd. (Fukuoka, Japan) was used for polarization of the free nitroxyl radical probes to enhance the MR image.

To demonstrate the feasibility of imaging in vitro plasma-generated ROS by DNP-MRI, a seven-well phantom (200 µl, 5.4-mm diameter, and 9-mm length) was used. Each tube was filled with a TEMPOL solution (1 mM TEMPOL + 2 mM GSH). Each well was irradiated with plasma for a different period, either in direct or remote mode, with the central well left unirradiated. The external magnetic field (B_0_) for EPR irradiation and MRI was fixed at 16 mT, with radiofrequencies of 458 MHz and 689 kHz, respectively. A 19-mm inner-diameter surface coil was used to perform DNP-MRI of the phantom with or without EPR irradiation. The DNP-MRI scanning conditions were as follows: power of EPR irradiation = 7 W, flip angle = 90°, repetition time (TR) × echo time (TE) × EPR irradiation time (TEPR) = 500 × 25 × 250 ms, 10 acquisitions, and 32 phase-encoding steps. After image reconstruction, the field of view (40 × 40 mm) was represented as a 64 × 64 matrix.

### Mouse skin CAP irradiation inflammation model

An electric clipper was used to clip the hair on the back of the mice from the neck to the tail followed by application of a commercial hair-removing cream, and the animals were left for 2 days before starting plasma irradiation. Under anesthesia with 2% isoflurane inhalation, mice (*n* = 4) were subjected to plasma irradiation into two different sites on their backs: one for the direct mode (5-min irradiation time) and the other for the remote mode (10-min irradiation time). A third site was used as a control and exposed to helium gas only. In our pilot experiments, a 5-min irradiation time was sufficient to induce clear skin damage in the direct mode, but not enough in the remote mode, which used 10 min.

The mice were irradiated with plasma at a distance of 5 mm between the plasma nozzle and skin. The animals were checked daily after irradiation, with photographs taken of their backs for 2 weeks, until the plasma-induced skin injury healed completely. ImageJ software (available online @ https://imagej.nih.gov/ij/) was used for measurement and analysis of the acquired image data from the in vivo plasma-induced skin lesions.

### In vivo DNP-MRI imaging of the skin tissue

A rectangular surface coil with a one-turn curve, prepared for skin imaging, was set and fixed on a special stage with a hole for TEMPOL administration. The mice were prepared as described previously^34^. Briefly, each mouse was placed on the stage in a supine position, which positioned the skin areas required for imaging in between the coil curve and stage hole, and the mouse was then secured to the stage.

The mice skins were irradiated by either plasma (remote mode) or helium gas as a control, as described above. At 24 h after irradiation, 2% isoflurane was used to anesthetize the mice. TEMPOL (150 µl of a 2.5-mM solution) was injected subcutaneously under the plasma-irradiated or control skin regions. DNP-MRI images were captured at 2, 4, 6, 8, 10, and 12 min after TEMPOL injection. The scanning conditions for the in vivo DNP-MRI experiment were as follows: power of EPR irradiation = 500 W, flip angle = 90°, TR × TE × TEPR = 1000 × 37 × 500 ms, coronal; number of averages = 2, slice thickness = 100 mm, phase-encoding steps = 32, field of view = 64 × 64 mm, and matrix size = 64 × 64. The MR images also were obtained without EPR irradiation. ImageJ software was used to calculate the image-enhancement decay rate of each mouse (*n* = 5/group) from the slope of the average image intensity in the region of interest equivalent to the enhancement by TEMPOL. A custom Excel microprogram was used to create a redox map from the slope of the image intensity of each pixel in four pharmacokinetic DNP images^34^.

### Conventional MRI anatomical imaging

Hair was removed from the mouse’s back as described above, and the skin was irradiated by the plasma remote mode at one site and helium gas only at the other site as a control. At 24 h after irradiation, the mouse was anesthetized with isoflurane (3% for induction and 2.0% for maintenance in 350 ml/min air), placed into a ^1^H MR volume coil in the prone position, and fixed using adhesive skin tape. Magnetic resonance images (sagittal plane of the whole mouse) and transverse planes of both the plasma-irradiated and control skin areas were obtained on a 1.5-T permanent magnet MRI system (Medalist) obtained from Japan Redox Ltd. (Fukuoka, Japan). T1-weighted proton images were obtained by use of a spin-echo sequence with a TR of 500 or 3000 ms, a TE of 10 or 40 ms, two or four accumulations, five 2-mm slices, and a 64 × 64 matrix.

### Histopathological examination

Plasma-irradiated and control skin samples were collected at 2 and 24 h after irradiation as follows: the mice were anesthetized with 4% isoflurane, euthanized, and their skin was surgically excised, spread by pins, and fixed in 10% formalin. Fixed tissues were embedded in paraffin, cut into 3-µm-thick sections, deparaffinized, and stained with hematoxylin and eosin. The sections were mounted, and a Keyence all-in-one fluorescence BZ-X800 microscope (Keyence, Osaka, Japan) was used to obtain images, and cellSens software (Olympus, Tokyo, Japan) was used for image analysis.

### Statistical analysis

All experiments were performed at least three times. Data are presented as the mean ± standard deviation. Student’s *t*-test (two-tailed) was performed to assess group differences in the means. The significance threshold was set at *P* < 0.05.

## Supplementary information

Supplementary Figure 1

## Data Availability

This study presents representative results from the ESR, in vivo DNP-MRI, MRI, and histopathology examinations. All other data and methods developed during this work are available on reasonable request from the corresponding author.
