# Supplementary material for: In vivo redox imaging of plasma-induced skin-inflammation in mice
Source: Npj Imaging. 2024 Aug 2;2:25. doi: 10.1038/s44303-024-00029-z (PMC12118652; doi:10.1038/s44303-024-00029-z)
Supplement: Supplementary file 1 — Supplementary Figure 1 [file 44303_2024_29_MOESM1_ESM.pdf]

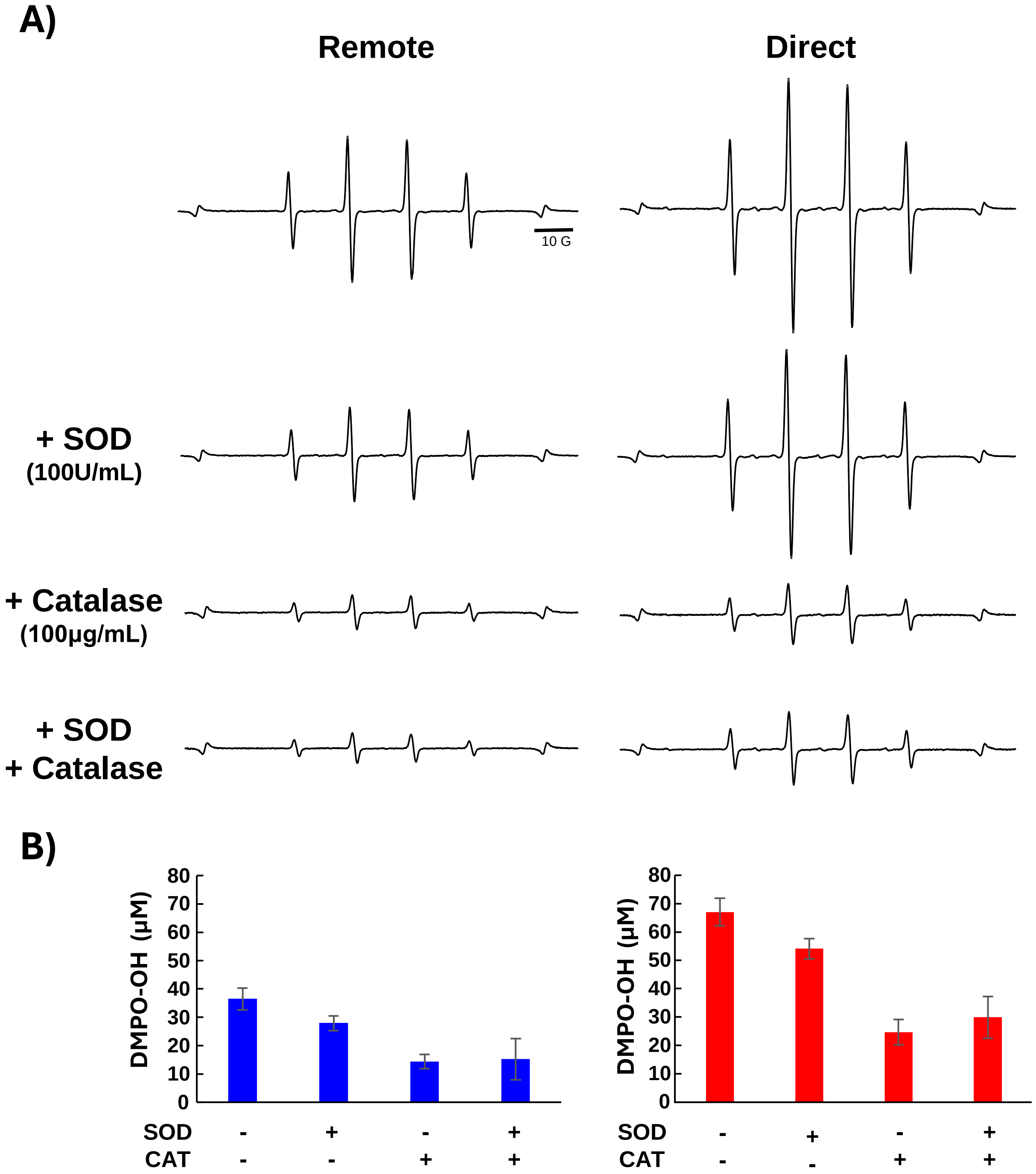

Supplementary Figure1: Effect of superoxide dismutase (SOD) and catalase on the plasma-induced EPR signal of DMPO-OH.
Plasma irradiation (remote or direct mode) was conducted on a 10 mM DMPO solution for 1 minute. DMPO solution was also mixed with 100 U/ml SOD, 100 µg/ml catalase, or both, and plasma irradiation was repeated. EPR signals were obtained immediately after exposure.
